# Supplementary material for: Patterns of Intron Gain and Loss in Fungi
Source: PLoS Biol. 2004 Nov 30;2(12):e422. doi: 10.1371/journal.pbio.0020422 (PMC532390; doi:10.1371/journal.pbio.0020422)
Supplement: Table S1 — Also available at http://genes.mit.edu/NielsenEtAl/. (4.3 MB ZIP). [file pbio.0020422.st001.zip › NielsenEtAl/html/128.html]

AN0387.1.NCU08626.1.MG06836.1.FG00797.1


```
 CLUSTAL W (1.82) Multiple Sequence Alignments - Introns Inserted


Sequence 1: MG06836.1	639 aa
Sequence 2: FG00797.1	635 aa
Sequence 3: NCU08626.1	642 aa
Sequence 4: AN0387.1	567 aa
Alignment Length: 663 aa
Number Identitical Residues: 251 aa
Alignment Score (without introns) 13952


MG06836.1 	--------MP~I~CLWHGYAPS1SAQRLLNSSIYLPKCSLGQFNTMAPRNQKRKAASPAG
NCU08626.1	----------~M~FRTIRTLTP~TTSVFPPTSLHFKLKTTMAPSKRKASAPPQTSHVNGN
FG00797.1 	MLGRSNSDIN2L0ILSLRVLQS~SLRSQLQTPRNTAFLSLAKFTTMPSKRLKRESSSPS-
AN0387.1  	----------~-~---------~-------------------------MRQKRK------
          	                                                    :       

MG06836.1 	SHGSDSKKIKTES--VEPISTSDPLRRPHTFYKESEENGIVLREFYPHEMSNERARAYNN
NCU08626.1	PSADKKRKTTTDAPPTNPNTSSDPLRAPHPFYKDSETHGIVLRKFYPHEMSNARAQAYND
FG00797.1 	-AGGTAKKAKTSK--MNGSGSSDALRRPHHKAQEAEENGIAIRKFYPAEMSNARARAYNN
AN0387.1  	-------------------LSNGADERYHPNAQQTADFGIVLRDFYPPEMSNARCEAYNS
          	                    :... .  *   :::   **.:*.*** **** *..***.

MG06836.1 	DEIPRPIEDLYQAMSQTHPIRQKVKVKGAVVHWFKMDLRVADNTALRLASEKAKSAGVPL
NCU08626.1	NELPRPIETLSAALAETAALRKSLPVRQAVVHWFKMDLRLHDNRSLWLASQKAKEAGVPL
FG00797.1 	NEIPRPIEDLVDALEVTAKERKSIDVKDAVVHWFKMDLRISDNRALALASDKAKEAGVPL
AN0387.1  	GILERPIESWQKAYTETSAQQNAMTANAAVVHWFKSDLRLHDNRALQMAYRVAREHKIPL
          	. : ****    *   *   :: : .. ******* ***: ** :* :*   *:.  :**

MG06836.1 	IALYTVSPKDWEAHLRAPVRVDFTLRSLEVLRQDLAKLDIPLHVETVDDRRAMKQRVLSL
NCU08626.1	ICLYVLSPEDLEAHLRAPIRVDFMLRTLEVLKTDLEDLGIPLWVETVEKRKEVPTKIKEL
FG00797.1 	IALYIISPQDYEAHLRAPVRIDFMLRTLSVIKEDLAKLDIPLYVETVEKRKHLPDRILEL
AN0387.1  	IGLYILSPEDLTAHLSSPARVDLTLRTLEQLKRDLGELDVPLYMETQACRKDIPRRIINL
          	* ** :**:*  *** :* *:*: **:*. :: ** .*.:** :**   *: :  :: .*

MG06836.1 	MEEWDASHLYANMEYEVDELRREANLVRLLAENGK----SMEVVHDTCVVPPGKLQTGTG
NCU08626.1	MKSWGASHLFCAMEYEVDELRREAKLVKLLAEGEKGEKMAADVVHDTCVVMPGALQSGSG
FG00797.1 	MDEWGASHLYANMEYEVDELRREAAMIKDFAENGK----AFEVVHDTCIVPPGELHTGAG
AN0387.1  	CQEWGANHLFANLEYEVDELRREAKLIRLCAENGIR----FETAHDMCVVPPGVLSSQQG
          	 ..*.*.**:. :*********** :::  **.        :..** *:* ** * :  *

MG06836.1 	RQYAVYSPWYRAWLKHLSGDPELLEPMEPPHKNPASAREKFSHLFDGKIPAAPKSKQMNE
NCU08626.1	GQYAVYSPWFRAWIKHIEENPECLEIYEKPGPNPPGTKEKHENLFACSIPEAPEGKRLRD
FG00797.1 	KQYAVYTPWFKTWIAHIHENLDLLELYEPPEKNPDSARKKFKKLFNAEIPEAPKSKRLGD
AN0387.1  	RQYAVYSPWFRSWLAFLNENPEYLEVSEDPTSNPGDARRYFKELFECPVPATPENKRFDD
          	 *****:**:::*: .:  : : **  * *  ** .::. ...**   :* :*:.*:: :

MG06836.1 	EEQQ-RLAAMWPAGEHEARKRLDAFIKRRIDGYAAKRNFPADDGTSSISVHLAAGTLSAR
NCU08626.1	DEKA-RYHSLWPAGEHEALKRLEKFCDEAIGKYAERRNIPAMQGTSNLSVHFASGTLSAR
FG00797.1 	EEKE-RLRSLWPCGEHEAKKRLDKFCEERIGNYHEKRNIPADAATSSLSVHLASGTISAR
AN0387.1  	VEKADRVRRAFPAGEHEGLRRLEEFLEKAT-AYDDERNFLSGETTSKLSPYFASGSLSAR
          	 *: .*    :*.****. :**: * ..    *  .**: :   **.:* ::*:*::***

MG06836.1 	TAVHLAHKASGTRKLDGGSQGIQTWISEVAWRDFYKHVLVNWPYVC2LTSAGSCPHSMNK
NCU08626.1	TAIRTARDRNNTKKLNGGNEGIQRWISEVAWRDFYKHVLVHWPYVC2----------MNK
FG00797.1 	TCVRTARDRNKTKKLNGGNDGIQTWISEVAWRDFYKHVLVNWPYVC2----------MNK
AN0387.1  	TAVAKAREAN-AGKISRGEAGYISWISEVAWRDFYKHVLVHWPFIC2----------MNK
          	*.:  *:. . : *:. *. *   ****************:**::*           ***

MG06836.1 	PFKPEYSNIAWSYDMEHFAAWCEGRTGFPIVDAAMRQLRGTGWMHNRCRMIVASFLAKDF
NCU08626.1	PFKPTYSNIEWSYNVDHFHAWTQGRTGFPIIDAAMRQVLSTGYMHNRLRMIVASFLAKDL
FG00797.1 	PFKPEYSNISWSYDNDHFAAWCEGRTGFPIVDAAMRQMNTIGYMHNRCRMIVASFLAKDL
AN0387.1  	CFKFEFTNIDWSYNNTHFEAWAAGKTGYPLVDAAMRQLHATAWMHNRCRMVVSSFLCKDL
          	 **  ::** ***:  ** **  *:**:*::******:   .:**** **:*:***.**:

MG06836.1 	LIDW~RMGERYFMENLVDGDFASNNGGWGFAASVGVDPQP1YFRVFNPLLQSEKFDPNGE
NCU08626.1	LVDW~RMGERYFMEHLIDGDFASNNGGWGFAASVGVDPQP~YFRVFNPLLQSEKFDPDGD
FG00797.1 	LLDW2RKGEKYFMEHLVDGDFASNNGGWGFSASVGVDPQP~YFRIFNPLLQSEKFDPDGD
AN0387.1  	LIDW~RRGERYFMEHLIDGDFASNHGGWGFGSSTGVDPQP~YFRIFNPIRQSERFDPDGE
          	*:** * **:****:*:*******:*****.:*.****** ***:***: ***:***:*:

MG06836.1 	YIRKWIPELKGI------KGKAIHDPYGRGCGVEAKK---KGYPKPLVDHKGCRERALKA
NCU08626.1	YIRKWVEELRDLPELKGGKGGEIHDPYGRGSEKVKKKLEEKGYPRPIVEHSGARDRALDA
FG00797.1 	YIRKWVPELKDL------DNKAIHDPYNRKAGPQAKK---KGYPEPIVTHKDCRERALAA
AN0387.1  	FIRRWVPELRGV------EGNAIHAPYERGAGDIARA---NGYPEPIVDHARQRGVALRK
          	:**:*: **:.:      ..  ** ** * .    :    :***.*:* *   *  **  

MG06836.1 	YKEGISKEI
NCU08626.1	YKRGLARDL
FG00797.1 	YKEGLAIDE
AN0387.1  	YKDAARGA-
          	** .
```
